# A functional 4-hydroxybenzoate degradation pathway in the phytopathogen *Xanthomonas campestris* is required for full pathogenicity

**DOI:** 10.1038/srep18456

**Published:** 2015-12-17

**Authors:** Jia-Yuan Wang, Lian Zhou, Bo Chen, Shuang Sun, Wei Zhang, Ming Li, Hongzhi Tang, Bo-Le Jiang, Ji-Liang Tang, Ya-Wen He

**Affiliations:** 1 State Key Laboratory of Microbial Metabolism, Joint International Research Laboratory of Metabolic & Developmental Sciences, School of Life Sciences & Biotechnology, Shanghai Jiao Tong University, Shanghai 200240, China; 2 State Key Laboratory for Conservation and Utilization of Subtropical Agro-bioresources, and College of life science and technology, Guangxi University, Nanning 530004, China

## Abstract

Plants contain significant levels of natural phenolic compounds essential for reproduction and growth, as well as defense mechanisms against pathogens. *Xanthomonas campestris* pv. *campestris* (*Xcc*) is the causal agent of crucifers black rot. Here we showed that genes required for the synthesis, utilization, transportation, and degradation of 4-hydroxybenzoate (4-HBA) are present in *Xcc*. *Xcc* rapidly degrades 4-HBA, but has no effect on 2-hydroxybenzoate and 3-hydroxybenzoate when grown in XOLN medium. The genes for 4-HBA degradation are organized in a superoperonic cluster. Bioinformatics, biochemical, and genetic data showed that 4-HBA is hydroxylated by 4-HBA 3-hydroxylase (PobA), which is encoded by Xcc0356, to yield PCA. The resulting PCA is further metabolized via the PCA branches of the β-ketoadipate pathway, including Xcc0364, Xcc0365, and PcaFHGBDCR. Xcc0364 and Xcc0365 encode a new form of β-ketoadipate succinyl-coenzyme A transferase that is required for 4-HBA degradation. *pobA* expression was induced by 4-HBA via the transcriptional activator, PobR. Radish and cabbage hydrolysates contain 2-HBA, 3-HBA, 4-HBA, and other phenolic compounds. Addition of radish and cabbage hydrolysates to *Xcc* culture significantly induced the expression of *pobA* via PobR. The 4-HBA degradation pathway is required for full pathogenicity of *Xcc* in radish.

The members of genus *Xanthomonas* are economically important bacterial pathogens. These infect at least 124 monocotyledonous and 268 dicotyledonous plants and cause severe damage^1^. *X. campestris* pv. *campestris* (*Xcc*), the causal agent of black rot in crucifers, is the producer of xanthan gum and thus is of great commercial and biotechnological application value^2^. In addition, *Xanthomonas* is also a scientifically important bacterial pathogen. *X. oryzae* pv o*ryzae* (*Xoo*), *X. campestris* pathovars, and *X. axonopodis* pathovars are currently recognized as three of the top 10 plant pathogenic bacteria in molecular plant pathology^3^.

A characteristic feature of *Xanthomonas* is the production of yellow, membrane-bound pigments called xanthomonadins^4^. These pigments are mixtures of unusual brominated, aryl-polyene esters^5^,^6^. A previous study conducted by Poplawsky and Chun^7^ has shown that xanthomonadin production in *Xanthomonas* is regulated by a diffusible factor (DF). Subsequent investigations showed that the DFs produced by *Xcc* and *Xoo* are 3-hydroxybenzoate (3-HBA) and 4-hydroxybenzoate (4-HBA)^8^,^9^. Our previous results showed that *Xcc* synthesizes 3-HBA and 4-HBA using the shikimate pathway product chorismate via the bifunctional chorismatase XanB2^10^. 3-HBA and 4-HBA are further used as intermediates for xanthmonadin synthesis via the *pig* cluster, and for CoQ8 biosynthesis, respectively^10^. Further genomic analysis revealed that *Xanthomonas* strains also contain the putative genes for the transportation and degradation of 3-HBA and 4-HBA (Fig. 1; Supplementary Fig. S1). These findings suggest that the phytopathogen *Xanthomonas* might have evolved an extensive ability to metabolize 3-HBA and 4-HBA. The mechanistic details and biological significance of this phenomenon remain to be elucidated.

Aromatic compounds constitute an important source of carbon and energy for soil-dwelling microorganisms and accumulate primarily as the result of the degradation of plant-derived molecules such as lignin^11^,^12^. Soil-dwelling microorganisms efficiently degrade a wide range of natural plant phenolic compounds, including 3-HBA and 4-HBA. The gentisate catabolic pathway has been described as the central route for 3-HBA degradation in some bacterial species^13^,^14^,^15^,^16^. Alternatively, 3-HBA could be degraded through the PCA catabolic pathway by the 3-HBA 4-hydroxylase, which is encoded by the *mobA* gene in *Comamonas testosteroni* KH122^17^. The PCA catabolic pathway, also called the PCA branches of the β-ketoadipate pathway, is a central catabolic route for aromatic compounds, which is widely distributed among taxonomically diverse bacteria and fungi^18^,^19^. PCA is a key central intermediate in bacterial degradation of diverse aromatic compounds, including 3-HBA, 4-HBA, and vanillate. PCA oxygenolytic ring-cleavage is catalyzed by PCA 3,4-dioxygenase (PcaGH) to generate 3-carboxy-cis,cis-muconate, which is converted into 4-carboxymuconolactone by 3-carboxy-cis,cis-muconate cycloisomerase (PcaB). 4-Carboxymuconolactone decarboxylase (PcaC) transforms 4-carboxymuconolactone into β-ketoadipate enol-lactone, which is then hydrolyzed by β-ketoadipate enol-lactone hydrolase (PcaD) into β-ketoadipate. The enzyme β-ketoadipate succinyl-CoA tranferase (PcaIJ) converts β-ketoadipate into b-ketoadipyl-CoA, which is finally transformed into succinyl-CoA and acetyl-CoA by β-ketoadipyl-CoA thiolase (PcaF)^19^. In some microorganisms, the PCA central pathway is involved in 4-HBA degradation. 4-HBA is hydroxylated by 4-HBA 3-hydroxylase, which is encoded by the *pobA* gene, to yield PCA in *Pseudomonas*, *Burkholderia*, *Acinetobacter calcoaceticus*, and *Cupriavidus*^19^,^20^,^21^,^22^. The resulting PCA is further metabolized via the PCA catabolic pathway.

The aims of this study were to characterize the 4-HBA degradation pathway and its biological significance in the model plant pathogen *Xcc*. This report described for the first time the genes and mechanism underlying 4-HBA degradation in plant pathogenic bacteria. This study demonstrated that the functional 4-HBA degradation pathway is required for full pathogenicity to Chinese radish and is probably involved in the plant-*Xanthomonas* interactions.

## Results

### *Xcc* genome contains a complete set of genes for 4-HBA metabolism

**Figure 1:**
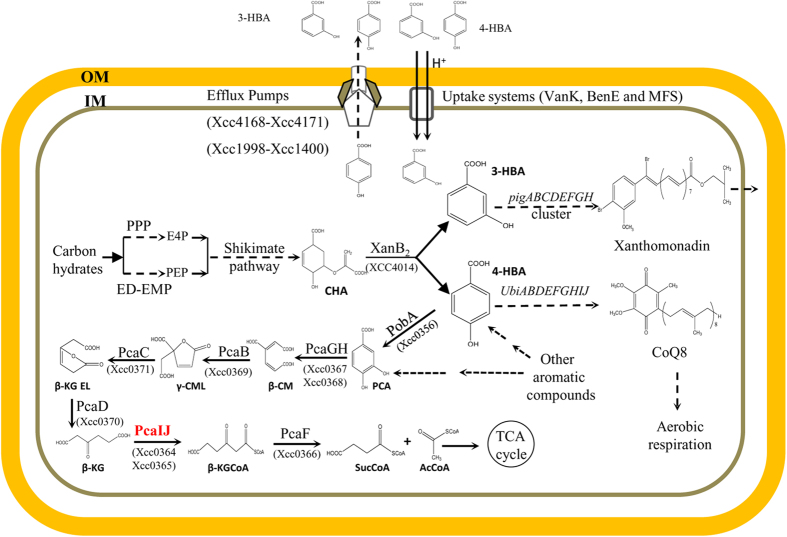
Schematic representation of a model of the synthesis, utilization, transportation, and degradation of 4-HBA in *Xcc*. OM, outer membrane; IM, inner membrane; PPP, pentose phosphate pathway; ED-EMP, Entner–Doudoroff pathway and Embden-Meyerhof-Parnas pathway; TCA, tricarboxylic acid cycle; E4P, erythrose-4-phosphate; PEP, phosphoenol-pyruvate; CHA, chorismate; 3-HBA, 3-hydroxybenzoate; 4-HBA, 4-hydroxybenzoate; PCA, protocatechuate; β-CM, β-carboxy-cis,cis-muconate; γ-CML, γ-carboxymuconolactone; β-KG EL, β-ketoadipate enol-lactone; β-KG, β-ketoadipate; β-KGCoA, β-ketoadipyl-CoA; SucCoA, succinyl-CoA; AcCoA, acetyl-CoA; PobA, 4-hydroxybenzoate-3-monooxygenase; PcaGH, protocatechuate 3,4-dioxygenase; PcaB, β-carboxy-cis,cis-muconate cycloisomerase; PcaC, γ-carboxymuconolactone decarboxylase; PcaD, β-ketoadipate enol-lactonase; PcaLM, β-ketoadipate succinyl-CoA transferase; and PcaF, β-ketoadipyl-CoA thiolase.

**Figure 2:**
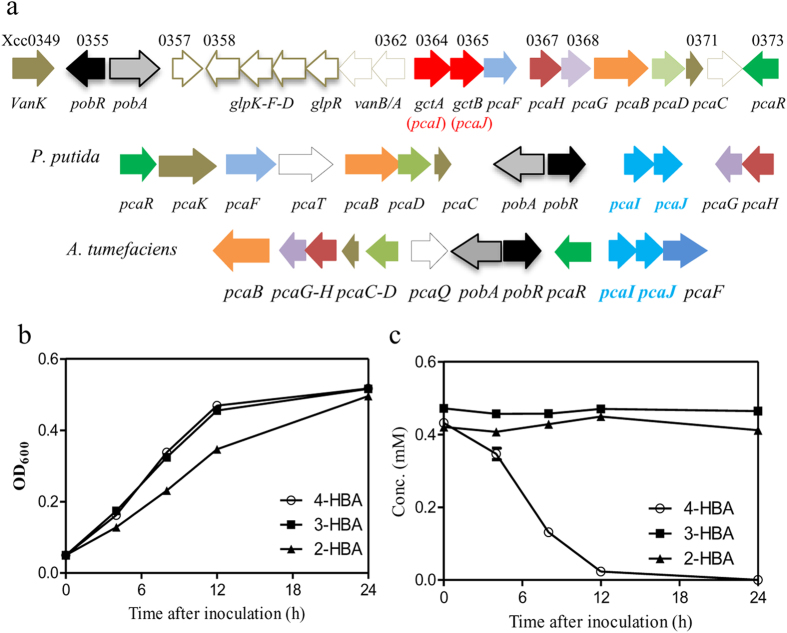
*Xcc* rapidly degrades 4-HBA. (**a**) The 4-HBA degradation gene cluster in *Xcc*, *Pseudomonas putida*, and *Agrobacterium tumefaciens*. (**b**) Growth time course of *Xcc* in the presence of 2-HBA, 3-HBA or 4-HBA in XOLN medium. (**c**) Time course of 2-HBA, 3-HBA, and 4-HBA levels in the supernatant of the XC1 culture during growth in XOLN medium.

In the present study, we conducted a global comparative genome analysis of *Xcc* wild-type strain ATCC33913 to identify the genes involved in 3-HBA and 4-HBA metabolism. In addition to the previously characterized genes for 3-HBA and 4-HBA biosynthesis and utilization, we also identified a range of putative genes for 3-HBA and 4-HBA uptake, efflux pumping, and degradation (Fig. 1 and Supplementary Fig. S1). Among these, the products of the cluster *Xcc4168*-*Xcc4171* are homologous to the previously identified 4-HBA efflux pump AaeXBA in *Escherichia coli*^23^ (Supplementary Fig. S1a). The gene cluster *Xcc1398*-*Xcc1400* is homologous to the 4-HBA exporter, PP1271-PP1273, which encodes a multidrug efflux MFS transporter in *Pseudomonas putida* S12^24^ (Supplementary Fig. S1b). The protein product of the gene *Xcc0349* is homologous to the characterized aromatic compound transporter BenK, VanK, or PcaK in *P. putida* or *Acinetobacter* sp. strain ADP1^25^,^26^,^27^. The genes *Xcc1685* and *Xcc4153* encode an MFS transporter and benzoate transporter, BenE, respectively (Supplementary Fig. S2b). In particular, the *Xcc* genome also contains a superoperonic gene cluster (*pca* cluster hereafter) that harbored the gene *pobA*, which encodes a 4-HBA 3-monooxygenase and those for the β-ketoadipate pathway identified in *P. putida* and *A. tumefaciens* (Fig. 2a). These findings suggest that *Xcc* is a strain with an extensive ability to metabolize 4-HBA.

### *Xcc* rapidly degrades 4-HBA

To further confirm whether the putative 4-HBA degradation pathway in *Xcc* was functional, 4-HBA was exogenously added into the XOLN cell cultures (OD_600_ = 0.1) at a final concentration of 0.5 mM. During growth, 4-HBA in the cultures was extracted and quantitatively analyzed by HPLC as previously described^10^. The results showed that the exogenous addition of 0.5 mM 4-HBA had little effect on *Xcc* growth (Fig. 2b; Supplementary Fig. S2b). The 4-HBA level in the culture rapidly decreased over time and a very low level of 4-HBA was detected in the culture after 12 h incubation (Fig. 2c). In contrast, when 3-HBA or 2-HBA was added to the same XOLN culture, its levels in the culture were relatively stable during growth (Fig. 2c), indicating that these were not degraded by *Xcc*.

### The *pca* locus is responsible for 4-HBA degradation in *Xcc*

The *pca* locus consists of a total of 19 genes ranging from *Xcc0355* to *Xcc0373* (a 20-kb gene cluster from position 426,627 to 446,943 in the chromosome of *Xcc* strain ATCC33913). Among these, the product of *Xcc0356* is highly homologous to PobA, which is a 4-HBA 3-monooxygenase that converts 4-HBA into PCA, whereas the product of *Xcc0355* is homologous to the regulator PobR in the environmental bioremediation strains *Pseudomonas*, *Burkholderia*, *Acinetobacter calcoaceticus*, and *Cupriavidus*^19^,^20^,^21^,^22^. The products of *Xcc366*-*Xcc0371* and *Xcc0373* are homologous to PcaFHGBDC and PcaR in the well-characterized β-ketoadipate pathway in the strains *Pseudomonas putida* KT2440, *A. tumefaciens*, and *Acinetobacter* sp. strain ADP1^19^,^21^. Therefore, *Xcc0355*, *Xcc0356*, *Xcc366*-*Xcc0371*, and *Xcc0373* were renamed accordingly as *pobA*, *pobR, pcaF*, *pcaH*, *pcaG*, *pcaB*, *pcaD*, *pcaC*, and *pcaR* in the present study.

**Figure 3:**
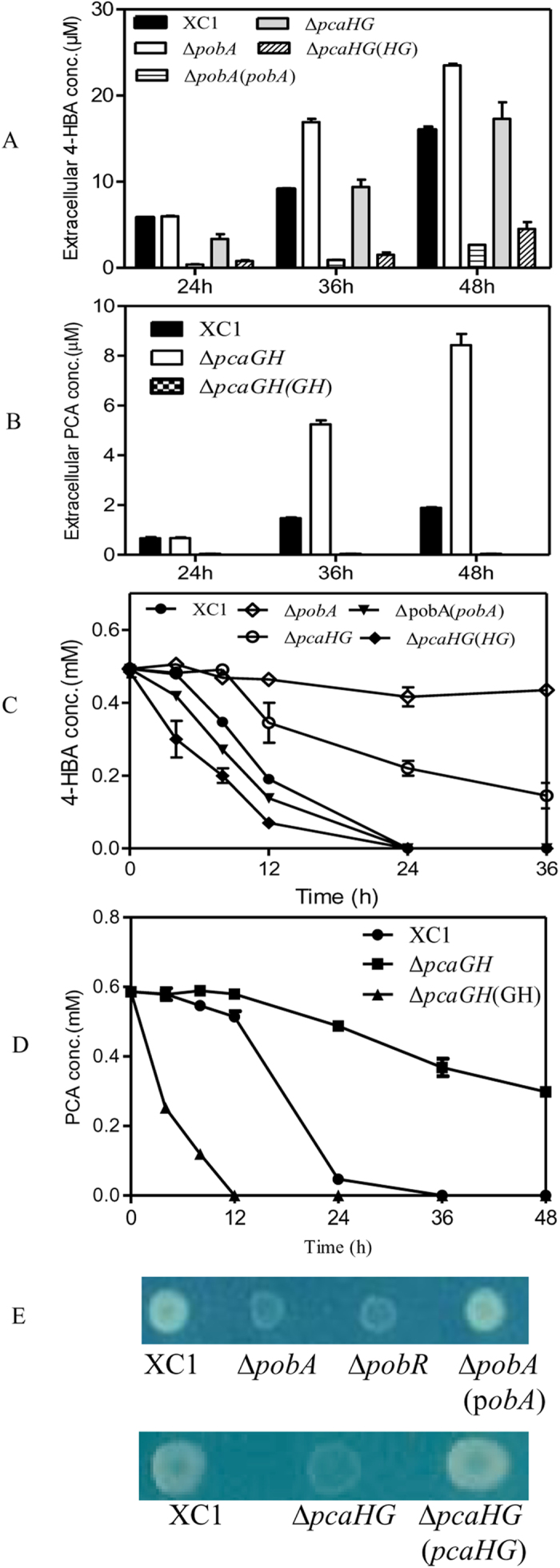
PobA and PcaGH are involved in 4-HBA and PCA degradation in *Xcc*. (**A**) Extracellular 4-HBA concentration of *Xcc* strains in NYG medium. (**B**) Extracellular PCA concentration of *Xcc* strains in NYG medium. (**C**) Time course of 4-HBA degradation of *Xcc* strains in XOLN medium with 0.5 mM 4-HBA. (**D**) Time course of PCA degradation of *Xcc* strains in XOLN with 0.5 mM PCA. (**E**) Growth of *Xcc* strains on an XOLN plate supplemented with 1.5 mM 4-HBA. Data are expressed as the means ± standard deviation of three independent assays.

Previous studies have shown that when *Xcc* is grown in a rich medium, it produces and secretes 3-HBA and 4-HBA into the supernatant^8^,^10^. We hypothesized that disruption of the 4-HBA degradation pathway promotes the production and secretion of 4-HBA. To test this hypothesis, *pobA* was deleted or overexpressed in *Xcc*. The resulting two strains, i.e., Δ*pobA* and Δ*pobA*(*pobA*), and the wild-type strain XC1 were respectively grown in NYG medium and the level of 4-HBA in the culture supernatant was determined. Our results showed that deletion of *pobA* led to significantly higher level of 4-HBA in the supernatant than that observed in the wild-type strain (Fig. 3a). Overexpression of *pobA* in the strain Δ*pobA* resulted in a decrease in 4-HBA production to a level lower than that observed in the wild-type (Fig. 3a). To further confirm the role of *pobA* in 4-HBA degradation in *Xcc*, the same three strains were grown in an XOLN liquid medium supplemented with 0.5 mM 4-HBA. Wild-type strain XC1 and strain Δ*pobA*(*pobA*) rapidly degraded 4-HBA, whereas strain Δ*pobA* almost lost its activity (Fig. 3c). *pobA* deletion or overexpression had no effect on Xcc cell growth in XOLN supplemented with 0.5 mM 4-HBA (Supplementary Fig. S2). Furthermore, strains XC1 and Δ*pobA*(*pobA*) showed normal growth on the XOLN plate supplemented with 1.5 mM 4-HBA, whereas strain Δ*pobA* presented poor growth (Fig. 3e), indicating that PobA was involved in 4-HBA degradation.

*pcaG* and *pcaH* encode the α- and β-subunits of protocatechuate 3,4-dioxygenase, which acts to convert PCA into β-carboxy-*cis,cis*-muconate^18^. Deletion of *pcaG* and *pcaH* significantly increased both exogenous 4-HBA and PCA production in the supernatant of NYG cultures, which was restored by overexpression of *pcaG* and *pcaH* in the mutant (Fig. 3a,b). When grown in XOLN medium with 0.5 mM 4-HBA or 0.5 mM PCA, strain Δ*pcaGH* almost lost its ability to degrade PCA or 4-HBA (Fig. 3c,d). Wild-type strain XC1 showed normal growth in the XOLN plate supplemented with 1.5 mM 4-HBA, whereas strain Δ*pcaGH* presented poor growth (Fig. 3e). These findings confirmed that *pcaG* and *pcaH* were also involved in 4-HBA and PCA degradation.

The *pca* locus also contains two genes, *Xcc0357* and *Xcc0372*, which encode hypothetical proteins, as well as the gene cluster *Xcc0358*–*Xcc0363* (Fig. 2a). The products of *Xcc0362* and *Xcc0363* are predicted to be responsible for vanillic acid metabolism. *Xcc0358*–*Xcc0361* was associated with glycerol uptake and catabolism. Deletion of these genes imparted minimal effects on exogenous 4-HBA levels, ability to degrade 4-HBA, and bacterial growth (Supplementary Fig. S3).

### *Xcc0364* and *Xcc0365* encode a different form of β-ketoadipate-CoA transferase

**Figure 4:**
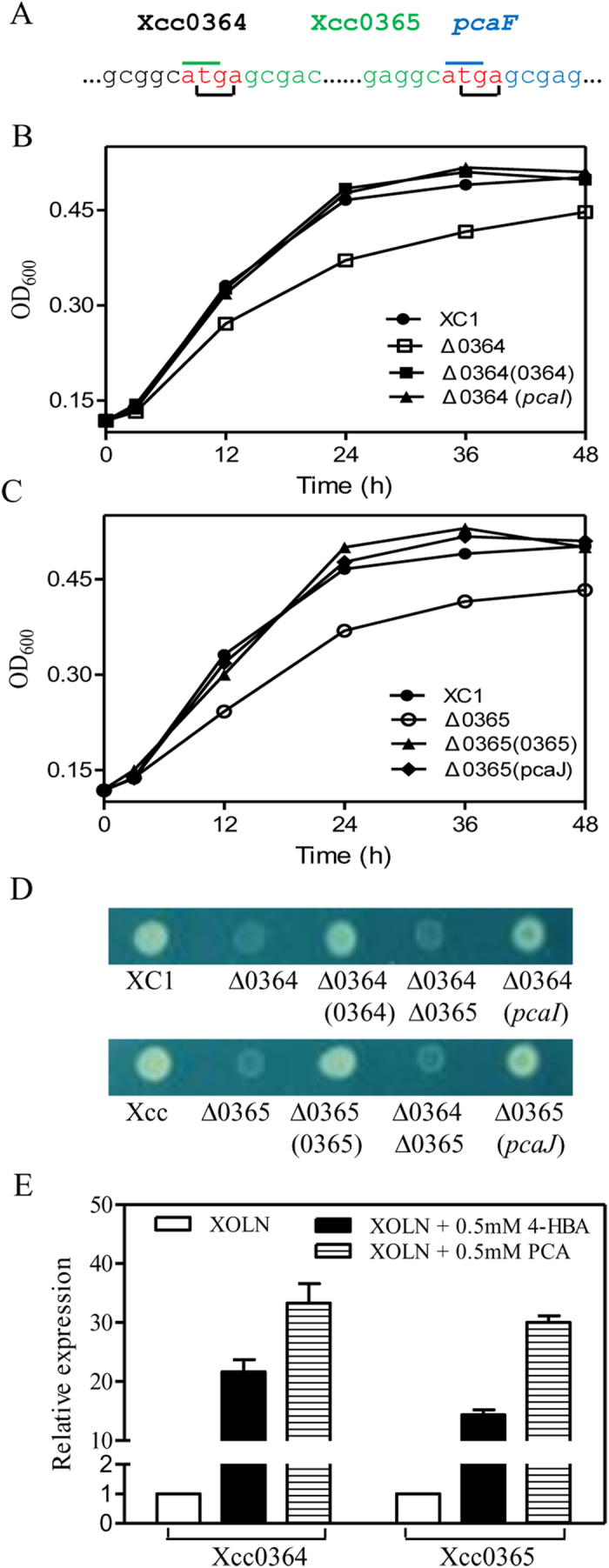
Xcc0364 and Xcc0365 are involved in 4-HBA degradation in *Xcc*. (**A**) Genetic organization of Xcc0364 and Xcc0365 in the *Xcc* genome. (**B**,**C**) Growth of *Xcc* strains in the XOLN medium supplemented with 1.5 mM 4-HBA. (**D**) Growth of *Xcc* strains on an XOLN plate supplemented with 2.5 mM 4-HBA. (**E**) Relative expression of Xcc0364 and Xcc0365 of XC1 strain in the presence of 0.5 mM 4-HBA or 0.5 mM PCA. Data are expressed as the means ± standard deviation of three independent assays.

In the β-ketoadipate pathway, β-ketoadipate succinyl-CoA transferase, which consists of a α-subunit (PcaI) and a β-subunit (PcaJ), is responsible for converting the β-ketoadipate into β-ketoadipyl-CoA^18^. In most β-ketoadipate pathway-containing bacterial species such as *A. tumefaciens* and *A. baylyi*, *pcaI*, *pcaJ*, and *pcaF* are usually transcribed within the same operon^28^. In the *pca* cluster of *Xcc*, genes encoding for β-ketoadipate succinyl-CoA transferase proteins (PcaIJ) were not detected (Fig. 2a). Two genes, *Xcc0364* and *Xcc0365*, which were originally annotated as glutaconate CoA transferase subunits A (*gctA*) and B (*gctB*), were localized upstream of *pcaF* (Fig. 2a). The coding sequences of Xcc0364, Xcc0365, and PcaF overlapped by three base pairs, respectively, in the chromosome (Fig. 4a), which suggested that these were organized as a single transcriptional unit and were functionally associated. Domain organization analysis showed that Xcc0364, Xcc0365, PcaI, and PcaJ belong to the same SugarP_isomerase superfamily and contained the same CoA_trans domain (Supplementary Figs S4, S5), further supporting our hypothesis. However, the low amino acid sequence similarity of Xcc0364 and Xcc0365 with PcaI and PcaJ in *A. tumefaciens* (PcaI, 18.7%; PcaJ, 19.4%) and *P. putida* (PcaI, 16.7%; PcaJ, 15.3%) prevented their annotation as orthologs of PcaI and PcaJ. In addition, signature sequences (glycine cluster and SENG motif, respectively) typically present in PcaI and PcaJ of many species were absent or modified in the products of *Xcc0364* and *Xcc0365* (Supplementary Figs S4, S5). These findings suggest that *Xcc0364* and *Xcc0365* might be encoding a different form of β-ketoadipate-CoA transferase.

To investigate whether *Xcc0364* and *Xcc0365* encode an β-ketoadipate succinyl-CoA transferase, we generated deletion and overexpression strains of the two genes, namely, Δ*Xcc0364* and Δ*Xcc0364*(*0364*), and Δ*Xcc0365* and Δ*Xcc0365*(*0365*). First, to determine whether β-ketoadipate accumulated from PCA metabolism in strains Δ*Xcc0364* or Δ*Xcc0365*, we performed the Rothera test, which detects the presence of β-ketoadipate and thus indicates whether PCA has been metabolized to this pathway intermediate^29^. The wild-type strain XC1 exhibited a Rothera-negative phenotype in the presence of 0.1 mM PCA, whereas strains Δ*Xcc0364* or Δ*Xcc0365* were Rothera-positive, indicating the accumulation of β-ketoadipate. The strains overexpressing *Xcc0364* or *Xcc0365* also resulted in a Rothera-negative phenotype. These results suggest that *Xcc0364* and *Xcc0365* are involved in β-ketoadipate metabolism in *Xcc*.

Second, the growth of all strains was compared in XOLN liquid or solid media supplemented with 4-HBA. Wild-type strain XC1 and strains Δ*Xcc0364* (*0364*) or Δ*Xcc0365* (*0365*) showed better growth than strains Δ*Xcc0364* or Δ*Xcc0365* in liquid XOLN medium with 1.5 mM 4-HBA (Fig. 4b,c) or XOLN plate with 2.5 mM 4-HBA (Fig. 4d). A previous study has shown that genes *pcaI* and *pcaJ* in *P. putida* encode the α and β subunits of β-ketoadipate succinyl-CoA transferase^18^. The present study showed that *pcaI*-overexpressing strain Δ*Xcc0364* followed a similar growth pattern to that of wild-type strain XC1 (Fig. 4c,d). Similarly, *pcaJ*-overexpressing strain Δ*Xcc0365* displayed a similar growth pattern as that of wild-type strain XC1 (Fig. 4c,d).

Finally, qRT-PCR analysis showed that addition of PCA or 4-HBA to the XC1 XOLN culture at a final concentration of 0.5 mM significantly induced the expression of *Xcc0364* and *Xcc0365* (Fig. 4e). Taken together, we concluded that *Xcc0364* and *Xcc0365* encode subunits of a new form of β-ketoadipate succinyl-CoA transferase, and these two genes were renamed *pcaI* and *pcaJ*, respectively. Further genomic assessment revealed that the homologs of *Xcc0364* and *Xcc0365* were not only present in most of the genomes of *Xanthomonas* species deposited in the NCBI microbe genome database, but also present in the genomes of *Lysobacter capsici*, *Pseudomonas aeruginosa* PAO1, *Pseudomonas knackmussii*, *Sinorhizobium meliloti*, and *Mesorhizobium loti*, with high amino acid identity (>60%) (Supplementary Figs S6, S7).

### *pobA* expression is significantly induced by 4-HBA via the transcriptional regulator PobR

**Figure 5:**
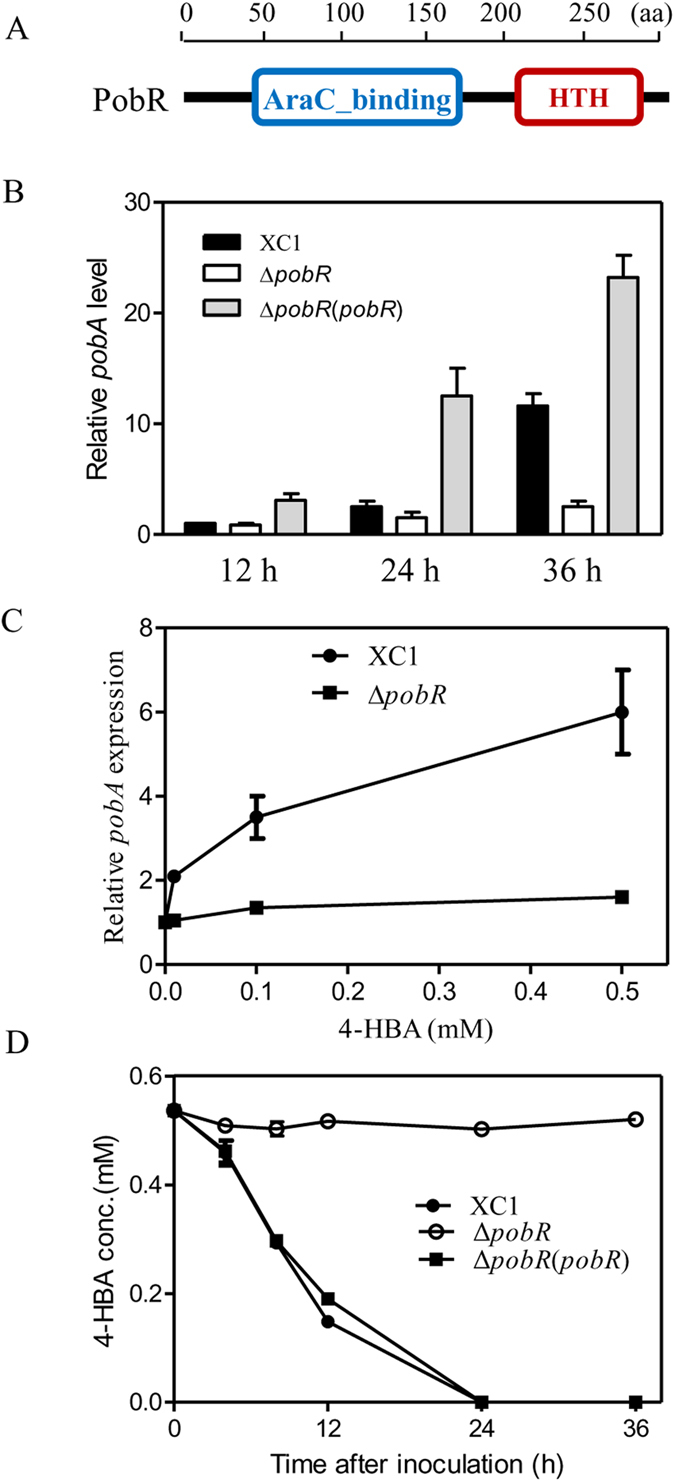
4-HBA induces the expression of 4-HBA degradation genes via the regulator, PobR. (**A**) Domain organization of PobR. (**B**) Time course of *pobA* expression in *Xcc* strains during growth. (**C**) Relative expression of *pobA* in strains XC1 and ΔpobR grown in the medium XOLN supplemented with 0.01 mM, 0.1 mM and 0.5 mM 4-HBA. (**D**) Time course of 4-HBA degradation of strains XC1, Δ*pobR*, and Δ*pobR* (*pobR*) in XOLN medium. Data are expressed as the means ± standard deviation of three independent assays.

The *pca* cluster in *Xcc* contains one gene, *Xcc0355*, which encodes an AraC-type transcriptional regulator, PobR (Fig. 5a). *pobR* is located adjacent to *pobA*, although its transcriptional orientation is in the opposite direction (Fig. 2a). PobR has been shown to be the activator for the 4-HBA degradation pathway in *Acinetobacter* sp. strain ADP1^30^. To study the effect of *pobR* on the expression of the 4-HBA degradation gene in *Xcc*, we generated *pobR* deletion and overexpression strains Δ*pobR* and Δ*pobR* (*pobR*). The expression pattern of *pobA* in *Xcc* strains during growth in XOLN medium or XOLN medium supplemented with 4-HBA was determined by qRT-PCR analysis. When grown in XOLN medium, *pobA* expression was relatively low at 12 h and 24 h after inoculation, and significantly increased at 36 h after inoculation (Fig. 5b). Deletion of *pobR* significantly reduced the expression of *pobA* at 36 h after inoculation, whereas overexpression of *pobR* resulted in the upregulation of *pobA* (Fig. 5b). Addition of 4-HBA (0.1 mM or 0.5 mM) to the wild-type XC1 culture resulted in a 3.5 ∼ 6.0-fold increase in the expression of *pobA*, but not in the Δ*pobR* culture (Fig. 5c). Furthermore, our results showed that deletion of *pobR* almost abolished 4-HBA degradation activity, and overexpression of *pobR* in strain Δ*pobR* restored 4-HBA degradation activity to that of the wild-type level (Fig. 5d). These findings suggest that 4-HBA via the activator PobR induced the expression of *pobA*.

### Radish and cabbage hydrolysates induce *pobA* expression

**Figure 6:**
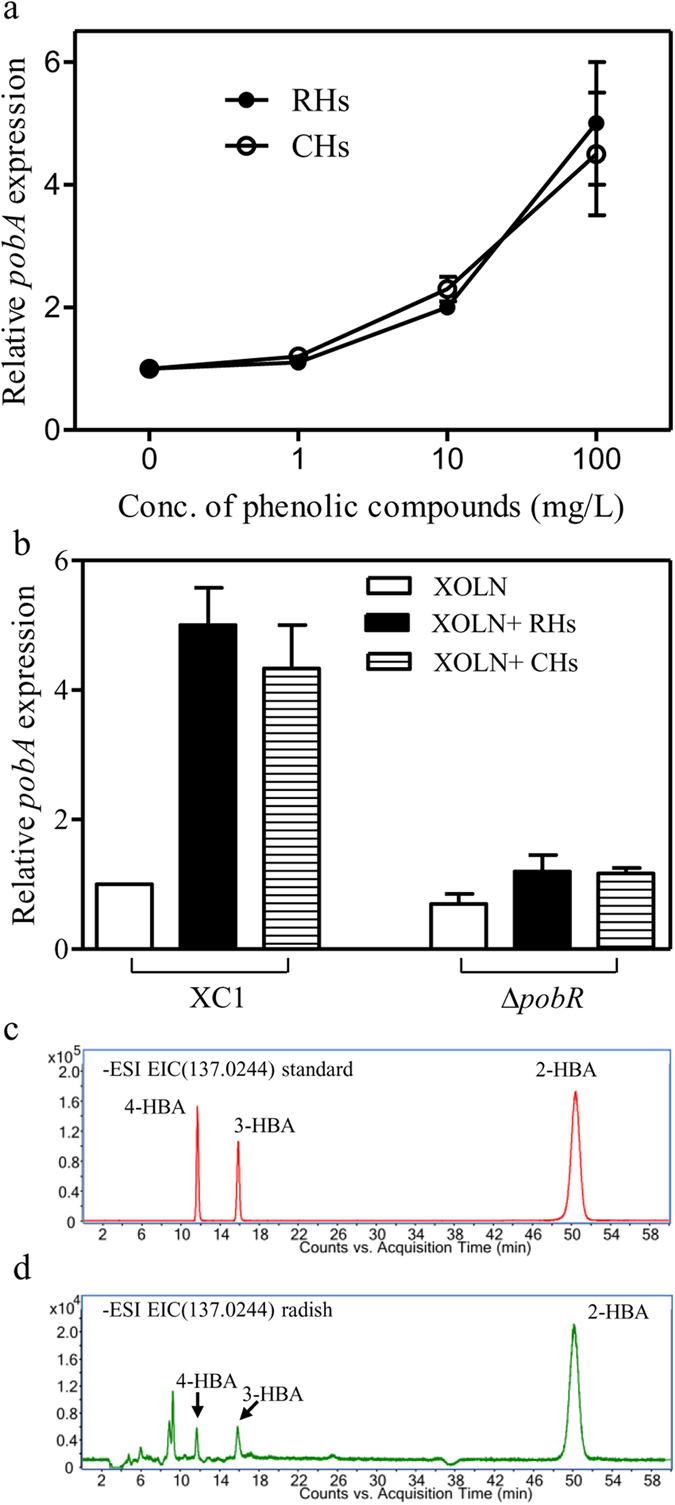
Relative expression of *pobA* in the presence of radish hydrolates (RHs) or cabbage hydrolates (CHs). (**a**) The dose-dependent *pobA* expression in the wild-type strain XC1 in the presence of 0.1–100 mg/L phenolic compounds. (**b**) The relative *pobA* expression in the strains of XC1 and Δ*pobR*. (**c**) The extracted ion chromatograms of standards 2-HBA, 3-HBA and 4-HBA at 50 μM. (**d**) The extracted ion chromatograms of 2-HBA, 3-HBA and 4-HBA in radish hydrolysates. Total RNA was extracted from the cultures 3 h after addition of the hydrolates. The relative levels of *pobA* were determined by quantitative real-time RT-PCR. Data are expressed as the means ± standard deviation of three independent assays.

Plants contain significant levels of natural phenolic compounds that play essential functions in plant reproduction and growth, as well as defense mechanisms against pathogens^31^. Phenolic acids are a major class of phenolic compounds, which mainly include hydroxybenzoic acids (e.g., gallic acid, 4-HBA, PCA, vanillic acid, and syringic acid) and hydroxycinnamic acids (e.g., ferulic acid, caffeic acid, p-coumaric acid, chlorogenic acid, and sinapic acid)^32^. We assumed that the 4-HBA degradation pathway in *Xcc* plays a role in detoxifying phenolic metabolites in the host during the infection. To test this hypothesis, radish and cabbage hydrolysates were prepared as described in Materials and Methods. Based upon the Folin-Ciocalteu method, the phenolic concentration within the radish and cabbage hydrolysates were 42.3 mg/g dry weight and 54.2 mg/g dry weight, respectively. The hydrolysate samples were added to the *Xcc* culture (OD_600_ = 0.8) in XOLN medium at three final phenolic concentrations, 1 mg/L, 10 mg/L, and 100 mg/L. After incubation for 3 h, the cells were collected for *pobA* gene expression analysis by qRT-PCR. Addition of radish or cabbage hydrolysates had little effect on *Xcc* growth (data not shown). The addition of the hydrolates at 10 mg/L or 100 mg/L phenolic compounds to the wild-type XC1 culture elicited a clear dose-dependent response in *pobA* expression (Fig. 6a). In contrast, the addition of the hydrolysates to the Δ*pobR* cultures had little effect on *pobA* expression (Fig. 6b).

Furthermore, the phenolic compounds in radish and cabbage hydrolysates were extracted as previously described^8^. LC-MS analysis revealed that radish hydrolysates contain 2-HBA, 3-HBA, 4-HBA, and other uncharacterized phenolic compounds (Fig. 6c,d; Supplementary Fig. S9a). Based on the established standard curves (Supplementary Fig. S9b), the absolute concentration of 2-HBA, 3-HBA, and 4-HBA present in radish leaves was estimated to be 262 ng/g fresh weight, 114 ng/g fresh weight, and 122 ng/g fresh weight, respectively. Similar phenolic compounds pattern was also observed in cabbage hydrolysates (data not shown).

### 4-HBA degradation pathway is required for full pathogenicity in radish

In the present study, the production of virulence factors such as extracellular polysaccharide (EPS) and extracellular enzymes in mutant strains Δ*pobA*, Δ*pcaG*, and Δ*pcaI* were compared to those in wild-type strain XC1 using the rich medium NYG. Our results showed that deletion of *pobA*, *pcaGH*, or *pcaI* had minimal effects on the production of cellulase, amylase, protease, and EPS (Supplementary Fig. S8).

To determine the role of the 4-HBA degradation pathway on the pathogenicity of *Xcc*, mutant strains Δ*pobA* and Δ*pcaGH* were inoculated in Chinese radish. Our results showed that the lesion length of these mutant strains 2 weeks after inoculation ranged from 12.1 mm to 14.0 mm, which respectively were 15.0% to 26.5% less than the observed 16.5 mm in the wild-type strain XC1.

## Discussion

**Figure 7:**
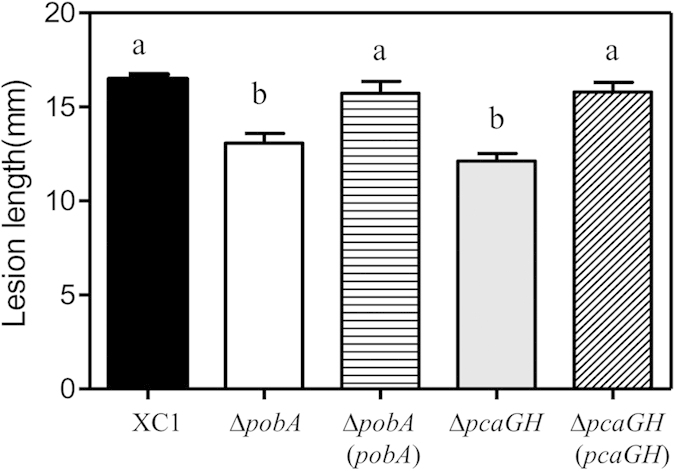
Virulence of *pobA* and *pcaGH* on Chinese radish. Virulence of the *Xcc* strains was tested by measuring lesion length after introducing bacteria into the vascular system of Chinese radish “Manshenhong” by leaf clipping. Values are expressed as the mean and standard deviation of triplicate measurements, each comprising 15 leaves. * and ** indicate significant differences between treatments (LSD at P = 0.05).

The present study demonstrated that the phytopathogen *Xcc* contains a functional 4-HBA degradation pathway, which consists of 4-HBA hydroxylase (PobA) and the PCA branches of the β-ketoadipate pathway. 4-HBA degradation activity has been experimentally shown in *P. putida*, *A. baylyi strain ADP1*, *A. tumefaciens*, and *C. necator* JMP134^30^,^33^,^34^,^35^. Generally, the genes for 4-HBA degradation are organized, function, and are regulated in *Xcc* in a manner similar to those of the above strains, in particular, to that previously described in *A. tumefaciens*. However, the present study also revealed several unique features in the 4-HBA degradation mechanism in *Xcc*. First, the 4-HBA degradation genes in *Xcc* are organized in a more complicated superoperonic gene cluster. In *A. tumefaciens*, the two *pca* operons were clustered in close proximity, flanking the putative *pobA* gene (Fig. 2a). In *P. putida*, the genes for 4-HBA degradation were dispersed in three discrete regions (Fig. 2a). In *Xcc*, the *pca* genes were located in two discrete operons, with the 4-HBA catabolic genes about 9 kb away and the glycerol and vanillic acid catabolic genes in the intervening regions (Fig. 2a). The multioperonal grouping of genes may reflect their acquisition by horizontal transfer, as well as their evolution in concert by sequence exchange^36^. Although the present study showed that the genes for glycerol and vanillic acid catabolism in the superoperonic cluster are not required for 4-HBA degradation in *Xcc* (Supplementary Fig. S3), its biological significance requires further investigations. Second, a new form of β-ketoadipate succinyl-CoA transferase was involved in 4-HBA degradation in *Xcc*, which will be discussed in the next section. Third, the present study, for the first time, has shown that the expression of 4-HBA degradation genes was significantly induced by the hydrolysates of the host plants (Fig. 7), suggesting that the 4-HBA degradation pathway was involved in the interaction between the plant and *Xanthomonas*.

In bacterial species such as *P. putida*, *A. baylyi*, *A. tumefaciens*, and *B. japonicum*, the transfer of CoA to β-ketoadipate is catalyzed by β-ketoadipate succinyl-CoA transferase (PcaIJ). In the present study, by combining the Rothera test, expression profiles, and genetic data, we demonstrated that Xcc0364 and Xcc0365 have similar activity to PcaIJ and were required for 4-HBA and β-ketoadipate degradation in *Xcc*. Although the products of *Xcc0364*/*Xcc0365* shared limited amino acid sequence identity to that of PcaIJ of *A. tumefaciens* and *P. putida* (Supplementary Figs S5, S6), these were highly homologous to those of SMB20587 (67%) and SMB20588 (60%), respectively, in *S. meliloti*. The latter two have been purified and shown to have β-ketoadipate succinyl-CoA transferase activity *in vitro*^11^. These findings strongly support that *Xcc0364* and *Xcc0365* encode the same form of β-ketoadipate succinyl-CoA transferase in *S. meliloti*. Therefore, the present findings are in good agreement with the previous assumption that at least two forms of β-ketoadipate succinyl-CoA transferase are present in bacteria^11^. The first one is present in the bacterial species such as *A. baylyi*, *P. putida*, *A. tumefaciens*, and *B. japonicum*, whereas the other one is present in *Xanthomonas* sp., *Lysobacter* sp., *Pseudomonas aeruginosa*, *M. loti*, and *S. meliloti*. The biological significance of the presence of two forms of β-ketoadipate succinyl-CoA transferase remains to be explored. It appears that in the course of evolution, natural selection has caused the β-ketoadipate pathway to assume a characteristic set of features or identity in different bacteria^18^. The new form of β-ketoadipate succinyl-CoA transferase encoded by *Xcc0364* and *Xcc0365* is present in most of the phytopathogens *Xanthomonas*. Whether these are related to specific lifestyles of *Xanthomonas* deserves further investigation.

Plants contain significant levels of natural phenolic compounds that play essential functions in the plant reproduction and growth, as well as defense mechanisms against pathogens^31^. In response to pathogenic attack, diverse broad spectrum antimicrobial substances are synthesized *de novo* by plants that accumulate rapidly at areas of pathogen infection. They may puncture the cell wall, delay maturation, disrupt metabolism or prevent reproduction of the pathogen in question^37^. Among these, glucosinolates and phenolics are well-known pathogen-induced metabolites of *Brassicaceae* family^38^,^39^. In addition, phytopathogens like *Xcc* may be exposed to a large amount of natural phenolic compounds derived from cell wall degradation during an infection. As a vascular pathogen, *Xcc* is normally restricted to the xylem tissues of infected plants. Within the xylem, *Xcc* multiplies and forms a microcolony, and then starts to produce various enzymes that would degrade the xylem walls for nutritional purpose^40^. The degradative enzymes not only cleave the cell wall to simple sugars, but also release lignin, which, when hydrolyzed, forms various types of aromatics such as 4-HBA, PCA, ferulic acid vanillic acid, and p-coumaric acid^22^. Several of these aromatics have been shown to be inhibitory towards fermentative microbes^41^,^42^. Some of them may influence the pathogen’s virulence machinery For instance, the classic immune hormone salicylic acid (2-HBA) has been shown to reduce virulence of *A. tumefaciens* by inhibiting the VirA/VirG two-component system^43^. In the opportunistic pathogen *Pseudomonas aeruginosa*, 2-HBA has been reported to reduce the production of several virulence factors including motility, biofilm formation and quorum sensing signal production^44^. Therefore, the ability of *Xcc* to survive phenolic compound stress is of critical importance for its successful colonization of host plants. The present study showed that *Xcc* contains a functional 4-HBA degradation system, which is required for full pathogenicity in radish (Fig. 7). The expression of the key gene *pobA* could be induced by 4-HBA or plant hydrolysates (Fig. 6). Therefore, the 4-HBA degradation system might be used to evade or subvert phenolic compound stress in *Xcc*. Similar results have also been reported in the Gram-positive *Arthrobacter* in the phyllosphere where the expression of *cph* genes for the degradation of pollutant 4-chlorophenol could be induced by natural phenolic compounds^45^. A similar 4-HBA degradation system is also present in other *Xanthomonas* species (Supplementary Figs S6, S7), suggesting that it could be a common strategy among phytopathogens. The detailed mechanisms on how 4-HBA degradation pathway contributes to the pathogenicity and plant-*Xanthomonas* interactions need to be further explored.

In addition to 2-HBA, 3-HBA and 4-HBA, plant phenolic compounds also include many other compounds like ferulic acid, vanillic acid, *p*-coumaric acid^32^. In bacteria, these compounds are initially transformed to a limited number of central intermediates, namely catechol and PCA. These intermediates are then channeled into two possible ring fission pathways, funneling them into the tricarboxylic acid cycle^13^,^22^. For example, ferulic acid is initially degraded to PCA via vanillic acid, whereas *p*-coumaric acid is degraded via 4-HBA in some Gram-negative bacteria^46^,^47^. These catabolic conversion steps required multiple genetic loci. The transformation of ferulic acid to vanillic acid involves an enoyl-CoA hydratase/aldolase, a vanillin dehydrogenase and a feruloyl coenzyme A synthase. Vanillic acid is then degraded to PCA by a demethylase encoded by two genes designated *vanA* and *vanB*^22^,^47^. The degradation of *p*-coumaric acid to 4-HBA also requires at least one locus that transforms ferulic acid to vanillic acid^47^. At lease two sets of *vanA* and *vanB* (*Xcc0361*-*Xcc0362*, *Xcc0296*-*Xcc0297*) were identified in the genome of *Xcc* strain ATCC33913. Interestingly, the former set is located within the *pca* cluster encoding 4-HBA degradation pathway (Fig. 2). These findings suggest that *Xcc* might degrade vanillic acid and other aromatic compounds via 4-HBA or PCA degradation pathway. Further genetic and functional identification of the molecular nature of diverse aromatic compounds degradation pathways in *Xcc* will not only help to elucidate the adaptation and virulence mechanism, but also provide a novel target for the development of *Xcc*-resistant crops.

## Methods

### Bacterial strains and growth conditions

The bacterial strains used in the present study are described in Supplementary Table S1. *Xcc* strain XC1 was grown in XOLN medium (5 g/L sucrose, 0.7 g/L K_2_HPO_4_, 0.2 g/L KH_2_PO_4_, 1 g/L (NH_4_)_2_SO_4_, 0.1 g/L MgCl_2_·6H_2_O, 0.01g/L FeSO_4_·7H_2_O, and 0.001 g/L MnCl_2_·4H_2_O, pH 7.15) or NYG medium (5 g/L peptone, 3 g/L yeast extract, and 2 g/L glycerol, pH 7.0) at 28 °C. *E. coli* strains were grown in LB medium at 37 °C. When required, rifampicin and kanamycin were added at final concentrations of 25 μg/mL and 50 μg/mL, respectively.

### Construction of in-frame deletion mutants and complementation analysis

The *Xcc* wild-type strain XC1 was used as parental strains for the generation of deletion mutants, as previously described^48^. The primers used are listed in Supplementary Table S2. For complementation analysis, the target gene was PCR amplified and cloned into the MCS site of the expression plasmid pBBR1MCS2. The resulting construct was transferred into *Xcc* by triparental mating.

### Extraction and quantitative analysis of 4-HBA and PCA by HPLC

4-HBA and PCA extraction and quantitative analysis were performed as previously described by Zhou *et al.*^10^. 4-HBA and PCA production was quantified using the peak area in HPLC elute. Commercially available 4-HBA and PCA (Sigma) were used as standards.

### Rothera test for the detection of β-ketoadipate

Rothera test was conducted following the method described by Holding and Collee^29^, with minor modifications. Briefly, an overnight NYG culture was centrifuged and washed with XOLN medium. Cells were subcultured into the XOLN medium supplemented with 0.1 mM protocatechuate for overnight incubation at 30 °C. Cells were centrifuged and resuspended in 0.02 M Tris-HCl (pH 8.0) to an optical density (OD) of 1.0. Toluene (0.5 mL) was added to 2 mL of resuspended cells, which was then incubated at 30 °C with shaking for 1 h. After shaking, 1 g of (NH_4_)_2_SO_4_ was added to the mixture and then vortexed. One drop of a fresh aqueous sodium nitroprusside (1%) solution was then added, followed by the addition of 1 drop of concentrated NH_3_ (29%), and the mixture was vortexed. Development of a purple color within 5 min following the addition of NH_3_ was considered as a positive indication for the presence of β-ketoadipate.

### Total RNA extraction and purification and qRT-PCR analysis

The total RNA of *Xcc* strains was extracted and purified using RNeasy Miniprep Kit (QIAGEN). Genomic DNA was removed by DNase I (QIAGEN). cDNA synthesis was conducting using PrimeScript RT Reagent Kit (TAKARA). RT-qPCR was performed in Mastercycler ep Realplex 4S (Eppendorf) with SYBR Premix EX Taq (TAKARA). Relative expression levels were calculated by using the 2^−Δ Δ CT^ method, and the gene *atpD* was used as reference to normalize all samples and replicates.

### Preparation of radish and cabbage hydrolysate samples and treatment of XC1 culture

A total of 1,000 g of radish (*Raphanus sativus* Manshenhong) or cabbage (*Brassica oleracea* L. Jingfeng) leaves were chopped into small pieces and blended in 200 mL of sterile water in an electric juicer (PHILIPS). The resulting samples were pretreated by adding NaOH at a final concentration of 1% (wt/vol) and by autoclaving at 121 °C for 15 min. After removing any remaining debris by passing the mixture through a filtration cloth, the filtrate was adjusted to a pH of 7.1 using hydrochloric acid (5 N) and lyophilized to generate a dry sample. The hydrolates were added to the XC1 cell culture at an OD_600_ = 0.8 at a range of final concentrations. After incubation for 3 h, the cells were collected for total RNA extraction and gene expression analysis. The phenolics present in the samples were quantified using the Folin-Ciocalteu reagent method^49^.

### Quantitative analysis of 4-HBA and other phenolic compounds in radish and cabbage hydrolysates via LC-MS

We followed the previously described method by He *et al.*^8^ to extract 4-HBA and other phenolic compounds in plant hydrolysates. The resulting residues were dissolved in 500 μl of methanol. A three-microliter aliquote of extracted sample was then injected into the ultra-performance liquid chromatography coupled with mass spectrometry apparatus (Agilent UPLC1290-TOF-MS6230) under the following conditions: Agilent Zorbax XDB C18 reverse-phase (5 μm, 4.6 × 150 mm) eluted with methonal with 0.1% formic acid and H_2_O with 0.1% formic acid (30:70) at 0.4 ml/min. The MS analysis was performed under negative mode with a scanning range of m/z = 100–1700. The specific pseudo molecular ion (M-H)^−^ of 2-HBA, 3-HBA, and 4-HBA were extracted at 137.0244. The retention time of 2-HBA, 3-HBA, and 4-HBA were 50.17 min, 15.88 min, and 11.68 min, respectively. The concentration of HBA molecules was quantified with a peak intensity (PI) of the specific extracted ion chromatogram (EIC) in the total ion chromatogram (TIC) according to the following formula: 2-HBA (μM) = 4.560 × 10^−6^ × PI + 0.404 with a R^2^ of 0.9994; 3-HBA (μM) = 2.643 × 10^−5^ × PI + 0.521 with a R^2^ of 0.9993; 4-HAB (μM) = 2.323 × 10^−5^ × PI + 0.181 with a R^2^ of 0.9998.

### Determination of extracellular enzyme activity and EPS production and virulence testing

Determination of extracellular enzyme activity and EPS production was performed as previously described^45^. *Xcc* virulence in Chinese radish was estimated by leaf clipping. Fresh cell cultures were used to inoculate at an OD_600_ of 0.01. The lesion length was scored 14 days after inoculation. Fifteen leaves from each tested strain were inoculated. Each strain was tested in at least three separate experiments.

## Additional Information

**How to cite this article**: Wang, J.-Y. *et al.* A functional 4-hydroxybenzoate degradation pathway in the phytopathogen *Xanthomonas campestris* is required for full pathogenicity. *Sci. Rep.* **5**, 18456; doi: 10.1038/srep18456 (2015).

## Supplementary Material

Supplementary Information
