# Supplementary material for: A functional 4-hydroxybenzoate degradation pathway in the phytopathogen Xanthomonas campestris is required for full pathogenicity
Source: Sci Rep. 2015 Dec 17;5:18456. doi: 10.1038/srep18456 (PMC4682078; doi:10.1038/srep18456)
Supplement: Supplementary Information [file srep18456-s1.pdf]

Supplementary Materials

Title: A functional 4-hydroxybenzoate degradation pathway in the phytopathogen *Xanthomonas campestris* is required for full pathogenicity

Authors: Jia-Yuan Wang, Lian Zhou, Bo Chen, Shuang Sun, Wei Zhang, Ming Li, Hongzhi Tang, Bo-Le Jiang, Ji-Liang Tang, Ya-Wen He

Contents

Supplementary Fig. S1

Supplementary Fig. S2

Supplementary Fig. S3

Supplementary Fig. S4

Supplementary Fig. S5

Supplementary Fig. S6

Supplementary Fig. S7

Supplementary Fig. S8

Supplementary Fig. S9

Supplementary Table S1

Supplementary Table S2

Supplementary Table S3

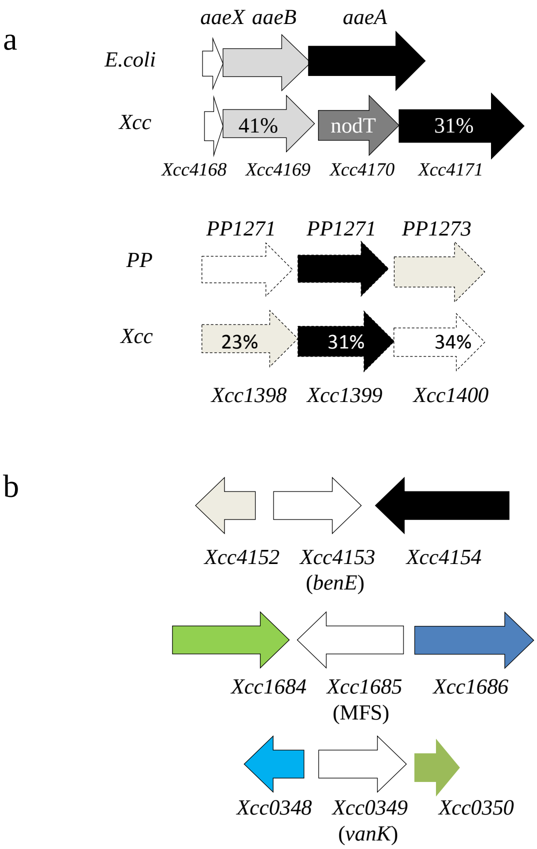

Supplementary Fig. S1 Putative genes or gene clusters for 4-HBA transportation in *Xanthomonas campestris* pv. *campestris*. (a) Putative gene clusters for 4-HBA efflux pumping; (b) Putative genes for 4-HBA uptake. The percentage indicates amino acid identity. All sequences were downloaded from the Microbial Genome Database of NCBI.

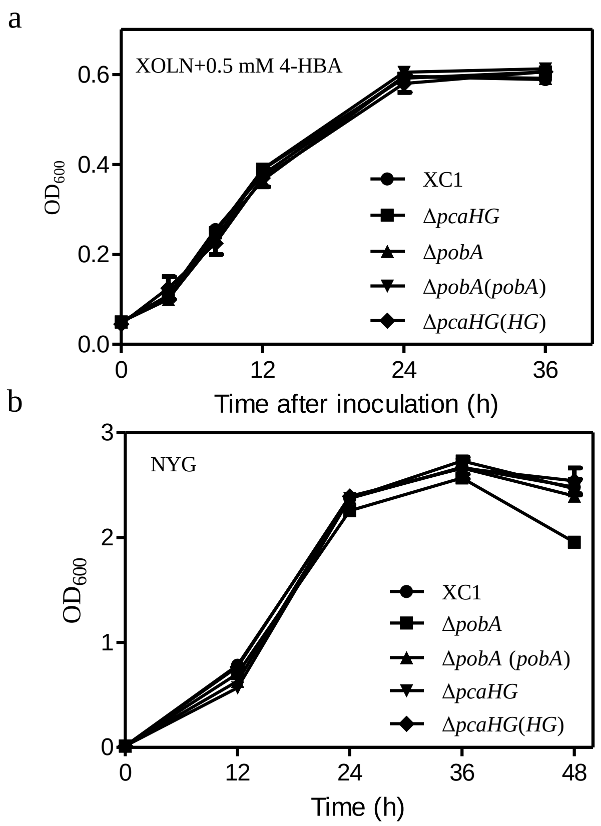

Supplementary Fig. S2 Growth of *Xcc* strains in (a) XOLN medium or (b) NYG medium supplemented with 0.5 mM 4-HBA.

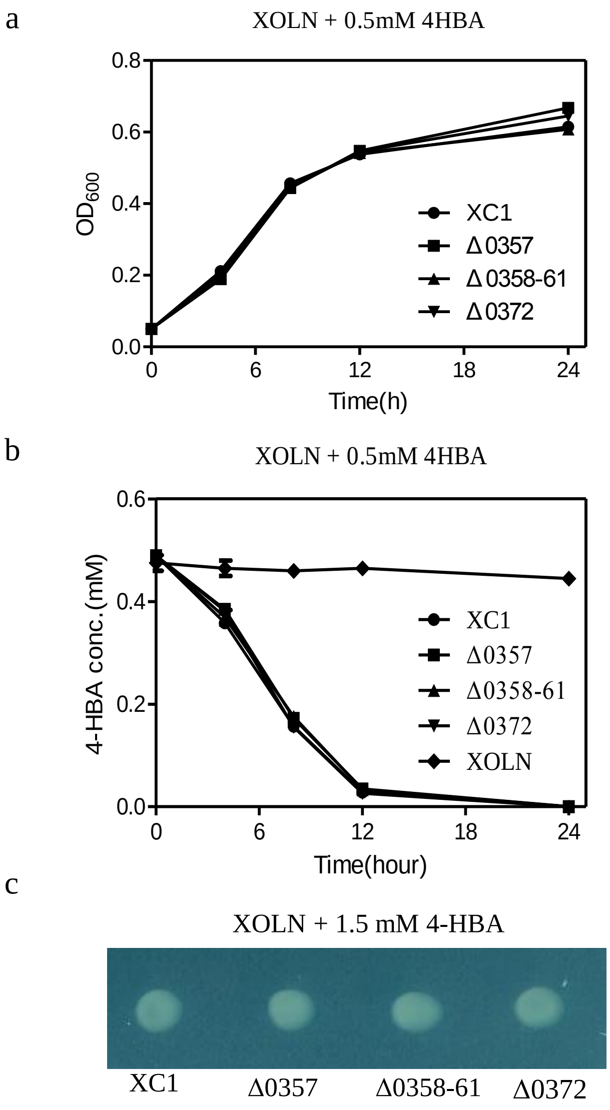

Supplementary Fig. S3 The genes Xcc0357, Xcc0358-61, and Xcc0372 are not required for 4-HBA degradation. (a) The growth of *Xcc* strains as determined at an optical density of 600 nm. (b) The 4-HBA levels in the cell cultures of *Xcc* strains during growth in XOLN medium. (c) Bacterial growth of *Xcc* strains on an XOLN plate supplemented with 1.5 mM 4-HBA.

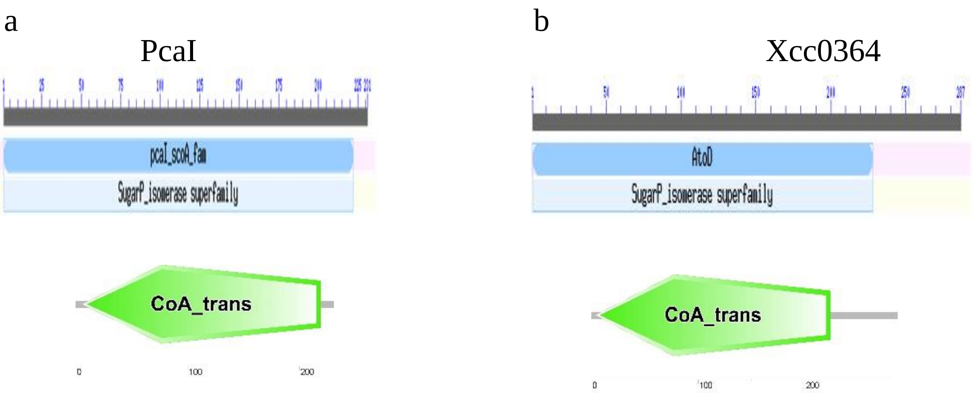

c

```
Xcc0364      MA-DIVSLSEAVAQLIADGDCVAMEGFTHL-IPHAAGHEVIRQRKRNLR-----------
pcaI         MINKTYESIASAVEGITDGSTIMVGGFGTAGMPSELIDGLIATGARDLTIISNNAGNGEI
             *  .  .   :..: *:**. : : **    :*    . :*    *:*
                               Glycine cluster

Xcc0364      -----------------LVRMTPDLIYDQLIGAGCASALRFSWGGNPGVGSLHRLRDAVE
pcaI         GLAALLMAGSVRKVVCSFPRQSDSYVFDELYRAGKIE-----------------------
                              : * : . ::*:*  **  .

Xcc0364      HGWPAPLQLREHSHADMANAYVAGASGLP-FAVLRGYV-----GSDLPKVNDSIRFLQCP
pcaI         --------LEVVPQGNLAERIRAAGSGIGAFFSPTGYGTLLAEGKETREID---------
                     *.   :.::*:   *..**:  *    **      *.:  :::

Xcc0364      YTGQTLATSPAVNPDVTVIHAQQADRRGNVLLWGIL-GVQKEAALAARKVIVTVEEIVDT
pcaI         --GRMYVLEMPLHADFALIKAHKGDRWGNLTYRKAARNFGPIMAMAAKTAIAQVDQVVEL
               *:  . .  :. *.::*:*::.** **:        .    *:**:..*. *:::*:

Xcc0364      LDAPPNACILPRWVVSAVCVVPGGAAPSYAHGYSARDNRFYLAWDAMARDRARFQAWIQA
pcaI         GELDPEHIITPGIFVQRVVAVTGAAASSIAKAV---------------------------
              :  *:  * *  .*. * .* *.** * *:.

Xcc0364      HILDTDDFAGFQRVYAGSQHAEAA
pcaI         ------------------------
```

Supplementary Fig. S4 Domain organization and multiple sequence alignment of Xcc0364 and PcaI. (a) Domain organization of PcaI in *Pseudomonas putida*. (b) Domain organization of Xcc0364 in *Xcc*. (c) Sequence alignment of PcaI and Xcc0364. Domain organization was conducted using the SMART program (http://smart.embl-heidelberg.de). Sequence alignment was conducted using the program Clustal Omega (http://www.ebi.ac.uk/Tools/msa/ clustalo/).

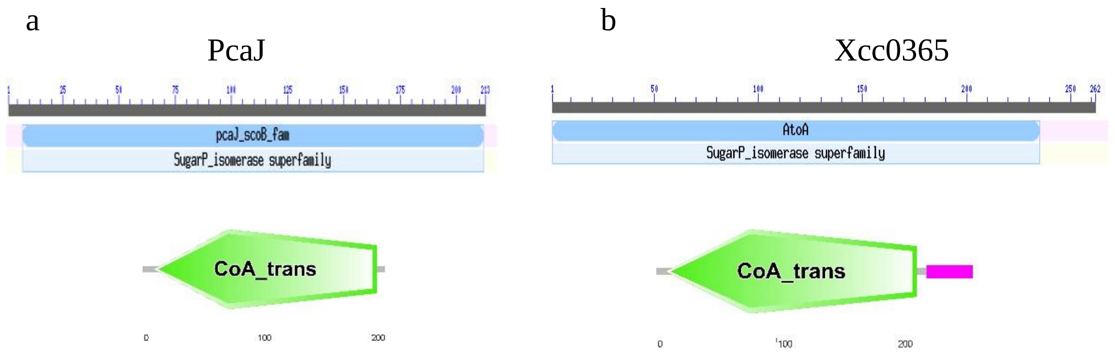

c

```
pcaJ          MTITKKLSRTEMAQRVAADIQEGAYVNLGIGAPTLVANYL---GDKEVFLHSENGLLGMG
Xcc0365       --MSDYSTNEMMTVAAARRLPDGAVCFVGIGLPSTAANLARLTHAPEVTLIYESGPIGAR
                ::.  :.  *:  .*  : :**   :*** *: .**        ** *  *.* :*
                                                                  SENG motif

pcaJ          PSPAPGEEDDDLINAGKQHVTLLTGGAFFHHADS-FSMMRGGHLDIAVLGAFQVSVKGDL
Xcc0365       PQVLPLSIGDGELAETAD--------TVVSTPEIFRYWLQGGRVDVGFLGAAQIDRHANL
              *.  * .  *  :    :        :..   :     ::**::*:..*** *:. :.:*

pcaJ          ANWH----TGAEGSIPAVGGAMDLATGARQVFVMMDHLTKTGESKLVP------------
Xcc0365       NTTVIGPYAAPKTRLPGAGGAPEIATSAKQVFIIMRQSTRSFVQTLDFITTVGHLDGGDA
               .      :. :  :*..*** ::**.*:***::* : *::  ..*

pcaJ          --ECTYPLTGIACVSRIYTDLAVLEVTPEGLKVV--EICADIDFDELQKLSGVPL-----
Xcc0365       RTRAGLPGAGP---TVVVTDLCVMEPDPVSRELTVTALHPGVTQAQVTAATGWAVRFAAT
                ..  * :*    : : ***.*:*  * . ::.   :   :   ::   :*  :

pcaJ          --------IK-------------------------
Xcc0365       LTETAAPTATELHALRALQARTAAAHGTQAAGAEA
                       .
```

Supplementary Fig. S5 Domain organization and multiple sequence alignment of Xcc0365 and PcaJ. (a) Domain organization of PcaJ in *Pseudomonas putida*. (b) Domain organization of Xcc0365 in *Xcc*. (c) Sequence alignment of PcaJ and Xcc0365. Domain organization was conducted using the SMART program (http://smart.embl-heidelberg.de). Sequence alignment was conducted using the program Clustal Omega (http://www.ebi.ac.uk/Tools/msa/ clustalo/).

XCC0364 homologues in Bacteria

a

```
Xcc0364  MADIVSLSEAVAQLIADGDCVAMEGFTHLIPHAAGHEVIRQRKRNLRLVRMTPDLIYDQL
SMB20587 MARIMSLAEAVEENVRDGDTVAMEGFTHLIPYAAGHEVIRQGRKDLFLVRMTPDILYDQL
         ** *:**:*** : : *** ***********:********* :::* *******::****

Xcc0364  IGAGCASALRFSWGGNPGVGSLHRLRDAVEHGWPAPLQLREHSHADMANAYVAGASGLPF
SMB20587 IGVGAARGMKFSWGGNPGVGSLHRFRDAVENQWPRPLEIEEHSHAAMANAYEAGAGNLPF
         **.*.* .::**************:*****. ** **::.***** ***** ***. ***

Xcc0364  AVLRGYVGSDLPKVNDSIRFLQCPYTGQTLATSPAVNPDVTVIHAQQADRRGNVLLWGIL
SMB20587 AAFRGYIGADLPKVNLNIRSVTCPFTGEVLAAVPAIRPDVTIIHAQRADRNGNVLIEGIV
         *.:***:*:****** .** : **:**:.**: **:.****:****:***.****: **:

Xcc0364  GVQKEAALAARKVIVTVEEIVDTLDAP-PNACILPRWVVSAVCVVPGGAAPSYAHGYSAR
SMB20587 GVQKEAVLAARRSIVTVEEIVDELSPPSPNSVVLPGWAVTAVAHVPGGAFPSYAHGYYPR
         ******.****: ********* *. * **: :** *.*:**. ***** ******* *

Xcc0364  DNRFYLAWDAMARDRARFQAWIQAHILDTD--DFAGFQRVYAGSQHAEAA
SMB20587 SNAFYIRWDEIARDRETFTAWIRENVLAAKPEDFARHAAKTAAKV----A
         .* **: ** :**** * ***: .:* :. *** . *.. *
```

b

Species	Homologues of Xcc0364	Amino acid identity (%)	NCBI access No.
Xac	XAC0364	92	AAM35256.1
Xcr	XCR_4151	99	AEL09008.1
Xff	XFF4834R_chr03 480	92	CDF60017.1
Xoc	XOC_4548	91	AEQ98605.1
Lysobacter capsici	AZ78_14030	79	EYR67734.1|
Pseudomonas aeruginosa	PA0226	73	AAG03615.1
Pseudomonas knackmussii	PKB_2952	72	CDF84299.1
Sinorhizobium meliloti	SMB20587	67	CAC49899.1
Mesorhizobium loti	MAFF_RS17350	58	BAB50895.1

Supplementary Fig. S6 Homologs of Xcc0364 in other bacterial species. (a) Multiple sequence alignment analysis of Xcc0364 and SMB20588. (b) Putative homologs of Xcc0364 in other bacterial species.

a

```
Xcc0365  -------MSDYSTNEMMTVAAARRLPDGAVCFVGIGLPSTAANLARLTHAPEVTLIYESG
SMB20588 MLEGKTKMTHFTPTEMMTVAAARALSNDDVCFVGIGAPSAACNVARLTNAPDITLIYESG
                *:.:: .********* * :  ******* **:*.*:****.**::*******

Xcc0365  PIGARPQVLPLSIGDGELAETADTVVSTPEIFRYWLQGGRVDVGFLGAAQIDRHANLNTT
SMB20588 TVGTKPDVLPLSIGDGELCDTALFTVSVPEMFRYWLQGGRITTGFLGGAQIDRFANLNTT
          :*::*:***********.:** . **.**:*********: .****.*****.******

Xcc0365  VIGPYAAPKTRLPGAGGAPEIATSAKQVFIIMRQSTRSFVQTLDFITTVGHLDGGDARTR
SMB20588 VVGPYDHPKVRLPGGGGAPEIASNCGRIFITMALTKRGFVERLPFVTSMGHGEGGNHRER
         *:***  **.****.*******:.. ::** *  :.*.**: * *:*::** :**: * *

Xcc0365  AGLPGAGPTVVVTDLCVMEPDPVSRELTVTALHPGVTQAQVTAATGWAVRFAATLTETAA
SMB20588 LGLTTSGPTRVITDLCILEPDPETKELTVVSMHPGVARDQIAGNCGWPIKFAETVIETPA
          ** : *** *:****::**** ::****.::****:: *::.  ** ::** *: ** *

Xcc0365  PTATELHALRALQARTAAAHGTQAAGAEA
SMB20588 PTETELVVLRDINARTKKAHKAAGKEAA-
         ** *** .** ::*** ** :  .  *
```

b

Species	Homologues of Xcc0365	Amino acid identity (%)	NCBI access No.
X. axonopodis pv. citri	XAC0365	89	AAM35257.1
Xcr	XCR_4150	100	AEL09007.1
Xff	XFF4834R_chr03490	89	CDF60018.1
Xoc	XOC_4547	90	AEQ98604.1
Lysobacter capsici	AZ78_14025	76	EYR67733.1
Pseudomonas aeruginosa	PA0227	72	AAG03616.1
Pseudomonas knackmussii	PKB_2951	74	CDF84298.1
Sinorhizobium meliloti	SMB20588	60	CAC49900.1
Mesorhizobium loti	MAFF_RS17345	63	BAB50894.1

Supplementary Fig. S7 Homologs of Xcc0365 in other bacterial species. (a) Multiple sequence alignment analysis of Xcc0365 and SMB20588. (b) Putative homologs of Xcc0365 in other bacterial species.

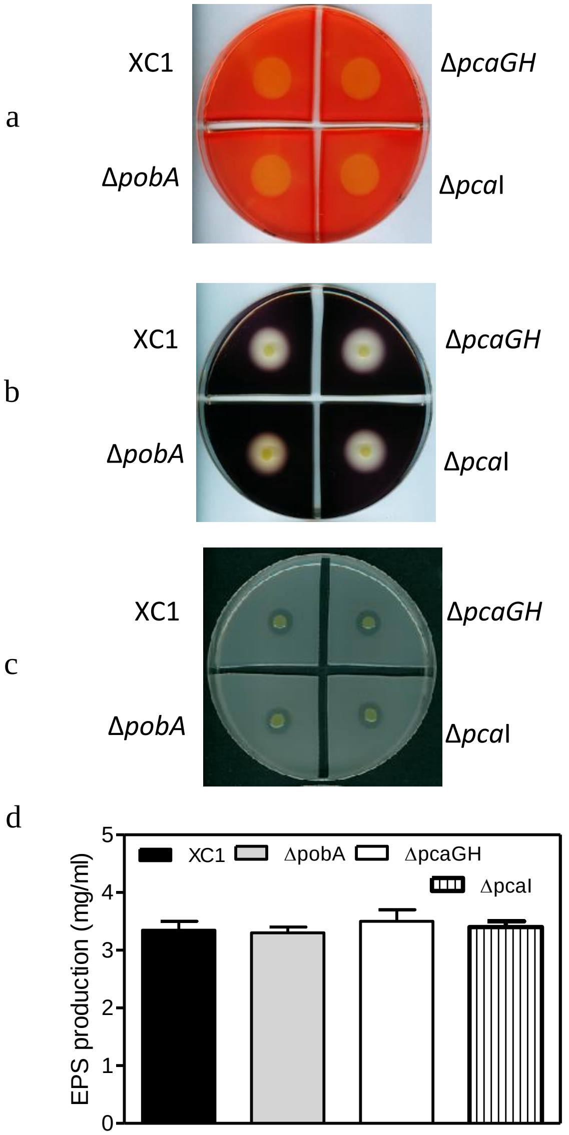

Supplementary Fig. S8 Production of extracellular enzymes and polysaccharide (EPS) in *Xcc* strains. (a) Cellulase activity. About 1 μL of the cell culture ($OD_{600} \approx 1.0$) was spotted onto NYG containing 0.5% CMC. (b) Amylase activity. *Xcc* strains were grown on an NYG plate supplemented with 0.1% soluble starch. (c) Protease activity. The strains were grown on an NYG plate supplemented with 1.0% skim milk. (d) EPS production in NYG medium.

a

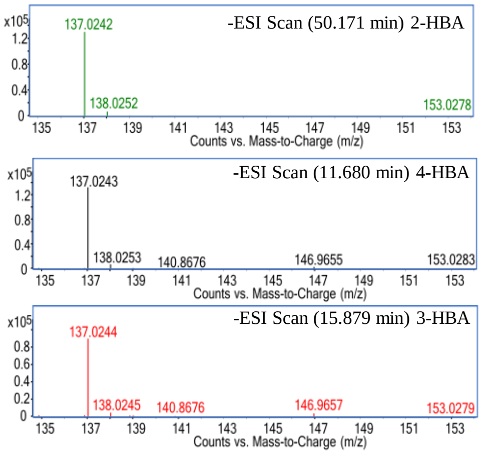

b

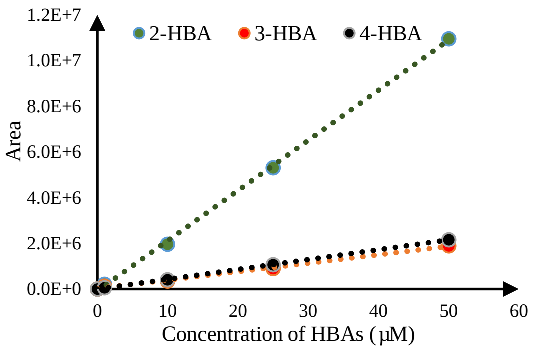

Supplementary Fig. S9 LC-MS analysis of radish hydrolysates. (a) Molecular weights of the putative 2-HBA, 3-HBA and 4-HBA detected in radish hydrolysates. (b) The established standard curves for determining the concentration of 2-HBA, 3-HBA and 4-HBA.

Supplementary Table S1. Bacterial strains used in this study

Strains	Relevant characteristics	Refernce/source
***Xcc* XC1**	Wild type, Rif^R	Laboratory collection
Δ0355	*xcc0355* in-frame deletion mutant of XC1	This study
Δ0356	*xcc0356* in-frame deletion mutant of XC1	This study
Δ0357	*xcc0357* in-frame deletion mutant of XC1	This study
Δ0358-61	*xcc0358, xcc0359, xcc0360, and xcc0361* in-frame deletion mutant of XC1	This study
Δ0364	*xcc0364* in-frame deletion mutant of XC1	This study
Δ0365	*xcc0365* in-frame deletion mutant of XC1	This study
Δ0364-65	*xcc0364* and *xcc0365* in-frame deletion mutant of XC1	This study
Δ0367-68	*xcc0367* and *xcc0368* in-frame deletion mutant of XC1	This study
Δ0370	*xcc0370* in-frame deletion mutant of XC1	This study
Δ0372	*xcc0372* in-frame deletion mutant of XC1	This study
Δ0373	*xcc0373* in-frame deletion mutant of XC1	This study
Δ0356C	Δ0356 mutant containing pBBR_0356 which contains full length *xcc0356*	This study
Δ0364C	Δ0364 mutant containing pBBR_0364 which contains full length *xcc0364*	This study
Δ0365C	Δ0365 mutant containing pBBR_0365 which contains full length *xcc0365*	This study
Δ0364(*pcaI*)	Δ0364 mutant containing pBBR_*pcaI* which contains full length *pcaI*	This study
Δ0365(*pcaJ*)	Δ0365 mutant containing pBBR_*pcaJ* which contains full length *pcaJ*	This study
Δ0367-68C	Δ0367-68 mutant containing pBBR_0367-68 which contains full length *xcc0367* and *xcc0368*	This study
P.putida		
KT2440	Wild type	Prof. Ping Xu lab collection
E.coli		
DH5α	F^-φ80*lac*Z△M15 *end*A1*hsd*R17 (r_k^- m_k^-) *sup*E44 *thi*$^{-1}$ *gyr*A96 △(*lacZYA-argF*)	Laboratory collection
S17-1	*recA pro* (RP4-2Tet::Mu Kan::Tn7)	Simon et al., 1983

Simon, R., Priefer, U., and Puhler, A. 1983. A broad host range mobilization system for *in vivo* genetic engineering: transposon mutagenesis in gram negative bacteria. Nat Biotech 1:784-791

Supplementary Table S2. Plasmids used in this study

Plasmids	Relevant characteristics	Refernce/source
pK18mobSacB	Suicide plasmid in XCC, Km^R	Schafer et al., 1994
pRK2013	ColE1 replicon TraRK+ Mob+; Helper plasmid for mobilisation of non-self-transmissible plasmids, Km^R	Figurski and Helinski, 1979
pBBR1MCS2	Plasmid for gene complementation, Km^R	Kovach et al.,1995
pK18-0355	Sucide construct for in-frame deletion of *xcc0355*	This study
pK18-0356	Sucide construct for in-frame deletion of *xcc0356*	This study
pK18-0357	Sucide construct for in-frame deletion of *xcc0357*	This study
pK18-0358-61	Sucide construct for in-frame deletion of *xcc0358, xcc0359, xcc0360, and xcc0361*	This study
pK18-0364	Sucide construct for in-frame deletion of *xcc0364*	This study
pK18-0365	Sucide construct for in-frame deletion of *xcc0365*	This study
pK18-0364-65	Sucide construct for in-frame deletion of *xcc0364* and *xcc0365*	This study
pK18-0367-68	Sucide construct for in-frame deletion of *xcc0367* and *xcc0368*	This study
pK18-0370	Sucide construct for in-frame deletion of *xcc0370*	This study
pK18-0372	Sucide construct for in-frame deletion of *xcc0372*	This study
pK18-0373	Sucide construct for in-frame deletion of *xcc0373*	This study
pBBR0356	Complementary vector containing whole length of xcc0356	This study
pBBR0364	Complementary vector containing whole length of xcc0364	This study
pBBR0365	Complementary vector containing whole length of xcc0365	This study
pBBR0367-68	Complementary vector containing whole length of xcc0367 and xcc0368	This study
pBBR-pcaI	Complementary vector containing whole length of pcaI	This study
pBBR-pcaJ	Complementary vector containing whole length of pcaJ	This study

Figurski, D.H., and Helinski, D.R. 1979. Replication of an origin-containing derivative of plasmid RK2 dependent on a plasmid function provided in trans. Proceedings of the National Academy of Sciences of the United States of America 76:1648-1652.

Kovach, M.E., Elzer, P.H., Hill, D.S., Robertson, G.T., Farris, M.A., Roop, R.M., 2nd, and Peterson, K.M. 1995. Four new derivatives of the broad-host-range cloning vector pBBR1MCS, carrying different antibiotic-resistance cassettes. Gene 166:175-176.

Schafer, A., Tauch, A., Jager, W., Kalinowski, J., Thierbach, G., and Puhler, A. 1994. Small mobilizable multi-purpose cloning vectors derived from the Escherichia coli plasmids pK18 and pK19: selection of defined deletions in the chromosome of Corynebacterium glutamicum. Gene 145:69-73.

Supplementary Table S3. Primers used in this study

Application	Primers	Sequence(5'-3')
In-frame deletion		
Δ0355	0355-F1	cccagccacccgaacggatacacc
	0355-R1	cgctacagccgcagatacattacgc
	0355-F2	tctgcggctgtagcggtggctgccgaccttggatttttc
	0355-R2	cacccacgacggcctgttctatgc
Δ0356	0356-F1	ggaattctcggcaaggctggcaaagacaaga
	0356-R1	aacctgggtgcgcatacggagt
	0356-F2	atgcgcacccaggttgcggcggtgttgcagcagtattc
	0356-R2	cgggatcccagcaacagggcgcgggagaac
Δ0357	0357-F1	gcccagccgcagccgttactacct
	0357-R1	cgctcgcccgctagacgctttg
	0357-F2	tctagcgggcgagcggacccgcgtgatccgtccaaga
	0357-R2	ccgggcatggcgaagaacacctat
Δ0358-61	0361-F1	cgacgcggcgaatgtgggtt
	0361-R1	cgccgcatacgccaggttctcc
	0358-F2	ctggcgtatgcggcgcgcggcaccagcaaggaacact
	0358-R2	ccgccaaccccaaccaccagaaa
Δ0364	0364-F1	ggaattccggccctgcgcgttgtagacc
	0364-R1	ggcgtgcgggatcaggtgggtaa
	0364-F2	ctgatcccgcacgccgcggaggcggcatgagcgacta
	0364-R2	cgggatcctggactgccgcatgatgatgaagac
Δ0365	0365-F1	ggaattcccccggacctgatttacgaccaac
	0365-R1	gccgatgccgacgaaacacacc
	0365-F2	ttcgtcggcatcggcggagccgaggcatgagcgagg
	0365-R2	cgggatcctccaggcgcggattgatgaagc
Δ0364-65	0364-F1	ggaattccggccctgcgcgttgtagacc
	0364-R1	ggcgtgcgggatcaggtgggtaa
	0365-F3	ctgatcccgcacgccggagccgaggcatgagcgagg
	0365-R2	cgggatcctccaggcgcggattgatgaagc
Δ0367-68	0367-F1	cgcgcgggtgctggggtttg
	0367-R1	gcgccgatagccgagcaggacat
	0368-F2	ctcggctatcggcgcctcgtgttcatgcgcgggctgt
	0368-R2	gtggcactgggcggcaatgacc
Δ0370	0370-F1	gcggcgatgtggtgctgctga
	0370-R1	gcgatgtgtcggcaactggagaaa
	0370-F2	ttgccgacacatcgcaacgacgtgctgctgggttttctc
	0370-R2	tcgggtgcgatgtctttgtgttga

Δ0372	0372-F1	gcctgcccgcccacactcg
	0372-R1	cggcagccgcgaccacaccac
	0372-F2	tggtcgcggctgccgctgctgctgcgcggtgtggatg
	0372-R2	cctggcggccctgggttatgtg
Δ0373	0373-F1	ggaattcacgccggcgtccaccagttg
	0373-R1	gggacgtttgcggcgggaagag
	0373-F2	cgccgcaaacgtcccctgcgtggcgaggtgattgc
	0373-R2	cgggatcctgttgtcggcgctggtgctgtatg
Complementation		
0356	0356-F	cccaagctttcggcaaggctggcaaagacaaga
	0356-R	cgggatcccgctcgcccgctagacgctttgac
0364	0364-F	cccaagcttcggccctgcgcgttgtagacc
	0364-R	gctctagagccgatgccgacgaaacacacc
0365	0365-F	cccaagcttatccgtttcctgcagtgcccctac
	0365-R	gctctagactggacagtgcgccgccgtaa
0367-68	0367-68-F	cccaagcttcgcgcgggtgctggggtttg
	0367-68-R	gctctagaccctggcgacctgtgcaaacaactac
pcaI	3951-F	ggaattcactgcatcgaaccactggaaagcg
	3951-R	cgggatcccggtgcgggagagctttttggtga
pcaJ	3952-F	ggaattccaacctggccgagcgtatccg
	3952-R	cgggatcctcgaagcggctgtaaaggtagtctcaag
qRT-PCR		
0364	RT0364-F	cttttcctggggcggcaatcct
	RT0364-4	ccatgtccgcgtggctgtgttc
0365	RT0365-1	caccccggagatctttcgctattgg
	RT0365-2	cagatgaccgacggtggtgatgaa
atpD	atpD-F	gcgatggtgtacggccagatgaa
	atpD-R	cggccagcgtgtagcggtagatgt
